# What the HLA-I!—Classical and Non-classical HLA Class I and Their Potential Roles in Type 1 Diabetes

**DOI:** 10.1007/s11892-019-1245-z

**Published:** 2019-12-09

**Authors:** Rebecca C. Wyatt, Giacomo Lanzoni, Mark A. Russell, Ivan Gerling, Sarah J. Richardson

**Affiliations:** 1 Institute of Biomedical and Clinical Science, University of Exeter Medical School, RILD Building, Barrack Road, Exeter, EX2 5DW UK; 2 Diabetes Research Institute, University of Miami – Miller School of Medicine, 1450 NW 10th Avenue, Miami, FL 33136 USA; 3 Department of Biochemistry and Molecular Biology, University of Miami – Miller School of Medicine, 1011 NW 15th Street, Miami, FL 33136 USA; 4 Department of Medicine University of Tennessee Health Science Center and VA Medical Center Research Service, 1030 Jefferson Avenue, Memphis, TN 38128 USA

**Keywords:** Type 1 diabetes, HLA-I, Non-classical HLA-I, HLA-E, HLA-F, HLA-G, Immune system

## Abstract

**Purpose of Review:**

Hyperexpression of classical HLA class I (HLA-I) molecules in insulin-containing islets has become a widely accepted hallmark of type 1 diabetes pathology. In comparison, relatively little is known about the expression, function and role of non-classical subtypes of HLA-I. This review focuses on the current understanding of the non-classical HLA-I subtypes: HLA-E, HLA-F and HLA-G, within and outside the field of type 1 diabetes, and considers the possible impacts of these molecules on disease etiology.

**Recent Findings:**

Evidence is growing to suggest that non-classical HLA-I proteins are upregulated, both at the RNA and protein levels in the pancreas of individuals with recent-onset type 1 diabetes. Moreover, associations between non-classical HLA-I genotypes and age at onset of type 1 diabetes have been reported in some studies. As with classical HLA-I, it is likely that hyperexpression of non-classical HLA-I is driven by the release of diffusible interferons by stressed β cells (potentially driven by viral infection) and exacerbated by release of cytokines from infiltrating immune cells.

**Summary:**

Non-classical HLA-I proteins predominantly (but not exclusively) transduce negative signals to immune cells infiltrating at the site of injury/inflammation. We propose a model in which the islet endocrine cells, through expression of non-classical HLA-I are fighting back against the infiltrating immune cells. By inhibiting the activity and function on NK, B and select T cells, the non-classical HLA-I, proteins will reduce the non-specific bystander effects of inflammation, while at the same time still allowing the targeted destruction of β cells by specific islet-reactive CD8+ T cells.

## Introduction

The human leukocyte antigen (HLA) gene family is the human form of the major histocompatibility complex (MHC). This gene family is clustered in a region of chromosome 6, and polymorphisms within this region confer approximately 50–60% of the overall risk of developing type 1 diabetes [1]. HLA genes encode for proteins that are key mediators of immune responses to pathogens, the development of self-tolerance and function as histocompatibility antigens in transplantation. A central role of these proteins is to present peptide antigens to the immune system, enabling recognition of non-self antigens and development of humoral, as well as cell-mediated immune responses. More than 200 genes form this complex, which is sorted into three groups: class I, class II and class III. HLA class II (HLA-II) risk alleles associated with type 1 diabetes confer the greatest genetic risk for this disease [2]. Certain HLA class I (HLA-I) alleles are also associated with type 1 diabetes, and there is some evidence of an association between single nucleotide polymorphisms (SNPs) in the class III region [1, 3].

HLA-II proteins are normally expressed by professional antigen-presenting cells (APCs), such as macrophages, dendritic cells and B cells. However, they can also be conditionally expressed by a wide range of cells, including epithelial, endocrine, endothelial and fibroblastic cells, in response to certain inflammatory mediators (e.g. interferon γ (IFNγ) and tumour necrosis factor α (TNFα) in islet cells) [4, 5]. HLA-II molecules, complexed with peptide antigens, are expressed by APCs on the cell surface and enable antigen presentation to CD4+ helper T cells. HLA-II molecules present peptide antigens that derive from proteins in the extracellular space. Extracellular proteins are internalised by APCs in endosomes, then converted by endosomal and lysosomal proteases into peptides; these peptides are loaded onto HLA-II molecules in a specialised class-II-loading vesicular compartment. The majority of HLA-II polymorphisms relate to the amino acid sequence of the peptide binding groove, which determines the repertoire of peptides that can be bound and displayed to T cells. Specific risk alleles for type 1 diabetes are implicated with the presentation of autoantigens targeted by islet autoimmunity.

The HLA-III region encompasses more than 60 genes, encoding proteins involved in the activation of complement, hormonal synthesis, inflammation and cell stress, extracellular matrix organisation and immunoglobulin superfamily members. Most class III proteins, however, have functions that are not directly implicated with the immune system [6].

HLA-I proteins (Table 1), in contrast, are expressed ubiquitously on all nucleated cells in the body for the presentation of intracellular self/non-self antigens to CD8+ cytotoxic T cell receptors and killer-cell immunoglobulin-like receptors (KIR). HLA-I molecules present intracellular antigens that originate from the cytoplasm. These are mostly proteins synthesised within the cell, but also proteins that enter the cytosol via phagosomes, and viral proteins. Peptides derived from cytosolic proteasome processing (from either constitutive proteasome or interferon-induced immuno-proteasome) are loaded on HLA-I molecules in the endoplasmic reticulum. HLA-I molecules can be split broadly into classical (HLA-A, B and C) and non-classical subtypes (including HLA-E, F, G and H). Non-classical HLA-I molecules are less polymorphic than their classical counterparts; they have the ability to present different types of intracellular antigens that are recognised by a different subset of innate immune receptors, and can be presented on the cell surface in response to different stimuli [7]. Non-classical HLA-I exert functions in both the innate and adaptive immune system, as discussed below. Importantly, when compared with classical HLA-I, non-classical HLA-I appear to have mostly inhibitory effects on immune cells, via interaction with inhibitory receptors. While most of these molecules are involved in antigen presentation and immunoregulatory functions, certain HLA-I exhibit non-immunological functions [8]. As an example, HLA-H (also known as HFE) is a protein implicated in iron metabolism: mutations in this gene are responsible for most cases of hereditary hemochromatosis, a disease of iron overload [9].

**Table 1:** Summary of non-classical HLA-I molecules (HLA-E, F and G), their expression in the pancreas and associations with type 1 diabetes

Non-classical HLA-I	Presented peptides and main function	Expression in the normal pancreas	Expression in T1D pancreas	Genetic associations with T1D
HLA-E	Displays a limited diversity of self-peptides, including leader sequences of HLA-I molecules Negatively regulates NK cells and a subset of T cells Interacts with inhibitory CD94/NKG2 receptors, found on most NK cells and a subset of T cells	Not present/very low-level expression in islets	Increased RNA and protein expression in insulitic islets Predominantly found in the α cells but also found in β cells Expression dependent on the presence of β cells	Limited evidence to suggest HLA-E*01:03 associated with younger age at onset SHLA-E*01:01 associated with older age at onset
HLA-F	Often exists as an open conformer but also binds a diverse range of peptides (> 2000) between 7 and 30 residues long Negatively regulates NK cells As an open conformer, interacts with inhibitory KIR3DS1 and KIR3DL2 receptors, found on NK cells When presenting a peptide, interacts with LIR1, also found on NK cells	Low-level expression in islets	RNA expression upregulated by islets from T1D donors Elevated protein expression in insulin-containing islets, primarily localised to the surface. Role in cross-presentation? Expression dependent on the presence of β cells	Unknown
HLA-G	Can display > 2200 peptides Negatively regulates NK, B and T cells Interacts with inhibitory ILT-2, ILT-4 and KIR2DL4 receptors, found on NK, B and T cells	Expressed by pancreatic islets and ducts Constitutively expressed by endocrine cells—low levels of β_2_M-free heavy chain proteins, mainly intracellular	Increased RNA and protein in islets Elevated protein expression in insulin-containing islets, found in both β and α cells Expression dependent on the presence of β cells	Strong association between deletion/deletion genotype of 14 bp of 3′ UTR and early age of onset Insertion allele associated with later age of onset

The structures of classical and non-classical HLA-I are similar: they are comprised of a peptide binding cleft (α1 and α2 domains) and an α3 domain, which forms a non-covalent association with β2 microglobulin (β_2_M) to stabilise the molecule. Although non-classical HLA-I molecules are structurally similar to classical subtypes, they have different affinities for peptide repertoires [7]. Moreover, soluble isoforms of HLA-G and HLA-E have been described [10, 11].

### Regulation of HLA-I

Stimulated transcriptional regulation of HLA-I genes usually falls under two main modules in the proximal promoter region: (a) the enhancer A (EnhA) and the interferon (IFN)-stimulated response element (ISRE) and (b) the SXY module. NOD-like receptor family CARD domain containing 5 (NLRC5) and signal transducer and activator of transcription 1 (STAT1) are important regulators of HLA gene expression as a part of coordinated immune responses to infections. In human embryonic kidney cells, NLRC5 binds and transactivates HLA class I gene promoters [12]. In vascular smooth muscle cells, the interaction of IFNγ and toll-like receptor 4 affected expression of a large number of STAT1-dependent genes including chemokine adhesion molecules and antiviral/antibacterial genes [13]. Whereas STAT1 regulates HLA-E, it does not appear to regulate HLA-G and HLA-F [14]. The main transcriptional regulators of HLA-G appears to be specificity protein 1 (Sp1), ISRE and SXY, whereas the main regulators of HLA-F are nuclear factor κB (NFκB), interferon regulatory factor 1 (IRF1) and class II, major histocompatibility complex transactivator (CIITA). CIITA also regulates HLA-E expression.

Although this all points to regulation by interferons, the differences highlight a potential for very complex and individualised responses to different infections and modes of interferon activation.

### HLA-I in Type 1 Diabetes

While evidence of aberrant HLA class II expression in the pancreatic islets of patients with type 1 diabetes is increasing [15], hyperexpression of HLA-I antigens in insulin-containing islets (ICIs) is now widely accepted as a defining hallmark of the disease. HLA-I hyperexpression is defined as dramatically elevated expression of HLA-I in all the islet cells (not just β cells), when compared to the surrounding acinar tissue of the same donor or other islets from individuals without type 1 diabetes.

HLA hyperexpression has been confirmed at both the RNA and protein levels in patients with recent-onset disease and in many patients who retain residual ICIs with < 10 years of diagnosis [16••]. Hyperexpression is observed in the ICIs of type 1 diabetes organ donors in the presence or absence of inflammatory infiltrates of the islets (termed ‘insulitis’) [17]. In support of this, studies examining insulitis in newly diagnosed type 1 diabetes donors over the age of 18 years demonstrate that only 25–30% of the ICIs have insulitis, yet all residual ICIs hyperexpress HLA-I [18, 19•]. It is possible that some of these islets have yet to experience insulitis, and thus, HLA-I hyperexpression might represent an earlier stage in the disease pathogenesis. It is hypothesised that HLA-I hyperexpression is primarily driven by the release of diffusible interferons by the β cells themselves [20•], which could derive from the sensing of an infection. Hyperexpression could be exacerbated following immune cell infiltration through the release of cytokines (including IFNγ) from the infiltrating cells. Importantly, once the β cells have been destroyed, the hyperexpression of HLA-I is lost [16••, 17]. The majority of studies in the field of type 1 diabetes have focused on the classical HLA-I (HLA-A, B and C), its accessory protein, β_2_M and transporter associated with antigen processing 1 (TAP1), which is involved in class I assembly. However, expression of select non-classical HLA-I molecules have been described in pancreatic islets, and aberrant regulation in the islets of individuals with type 1 diabetes has been reported [16••, 21]. However, much less is known about the function of these non-classical isoforms and the role they may play in type 1 diabetes. This will be the focus of the following review.

## Function of Non-classical HLA Class I

### HLA-E

HLA-E shares many structural features with classical class I molecules. It is assembled and regulated using common pathways and is ubiquitous throughout tissues, but is the least polymorphic of the HLAs and is transcribed at lower rates. Twenty-seven HLA-E alleles encode 8 proteins, according to the IMGT/HLA Database (http://hla.alleles.org/ April 2019). Under basal conditions, the HLA-E protein is present predominantly in immune cells and endothelial cells, and cell surface presentation of HLA-E requires the loading of one of a limited range of peptides [10]. Transcription of HLA-E is upregulated by IFNγ, mediated by an upstream STAT1 binding site. HLA-E transcription can be also be induced by CIITA through the SXY regulatory module, but it is not upregulated by NFκB or IRF1 [14]. Two known functional variants of this molecule are HLA-E*01:01 (HLA-E107R) and HLA-E*01:03 (HLA-E107G), differing by a single amino acid at position 107 (Arg/Gly). This change impacts the thermal stabilities of the HLA/peptide complexes and their length of interaction with cognate receptors, as well as expression levels [22]. It has been speculated that individuals carrying HLA-E*01:03 have high levels of the molecule with a greater affinity for viral antigens [23].

As stated above, a limited diversity of self-peptides can occupy the peptide binding groove of HLA-E. This includes the leader sequences of HLA-I molecules HLA-A, HLA-B, HLA-C and HLA-G. When these peptides bind HLA-E, the resulting complex is presented on the cell surface and can interact with inhibitory CD94/NKG2 receptors, located on most natural killer (NK) cells and on a subset of T cells. This is a critical mechanism of tolerance and self-surveillance. In tumour cells, for instance, the loss of classical HLA-I expression would provide a survival advantage, but the subsequent reduction in availability of leader peptides, along with the co-regulation, determines a decrease in HLA-E presentation: this removes signals that are inhibitory for NK cells, licensing them to kill the target tumour cells.

Micro-environmental stresses such as hypoxia and glucose deprivation can lead to upregulation of HLA-E [24]. An environment that impairs the peptide transporter TAP, which can be inhibited by a viral infection or in tumour tissue, can determine a reduction in availability of HLA-I leader peptides and can lead to an alternative repertoire of peptides being presented in the context of HLA-E; this can subsequently impact the outcome of the viral infection or the elimination of damaged or neoplastic cells [24–26]. In endothelial cells, the pro-inflammatory mediators TNFα, interleukin-1β and IFNγ can upregulate the cell surface expression of HLA-E and induce the release of soluble HLA-E. Upregulation of membrane-bound HLA-E protects activated endothelial cells from NK cell lysis, whereas soluble HLA-E protects bystander cells [10].

**Fig. 1:**
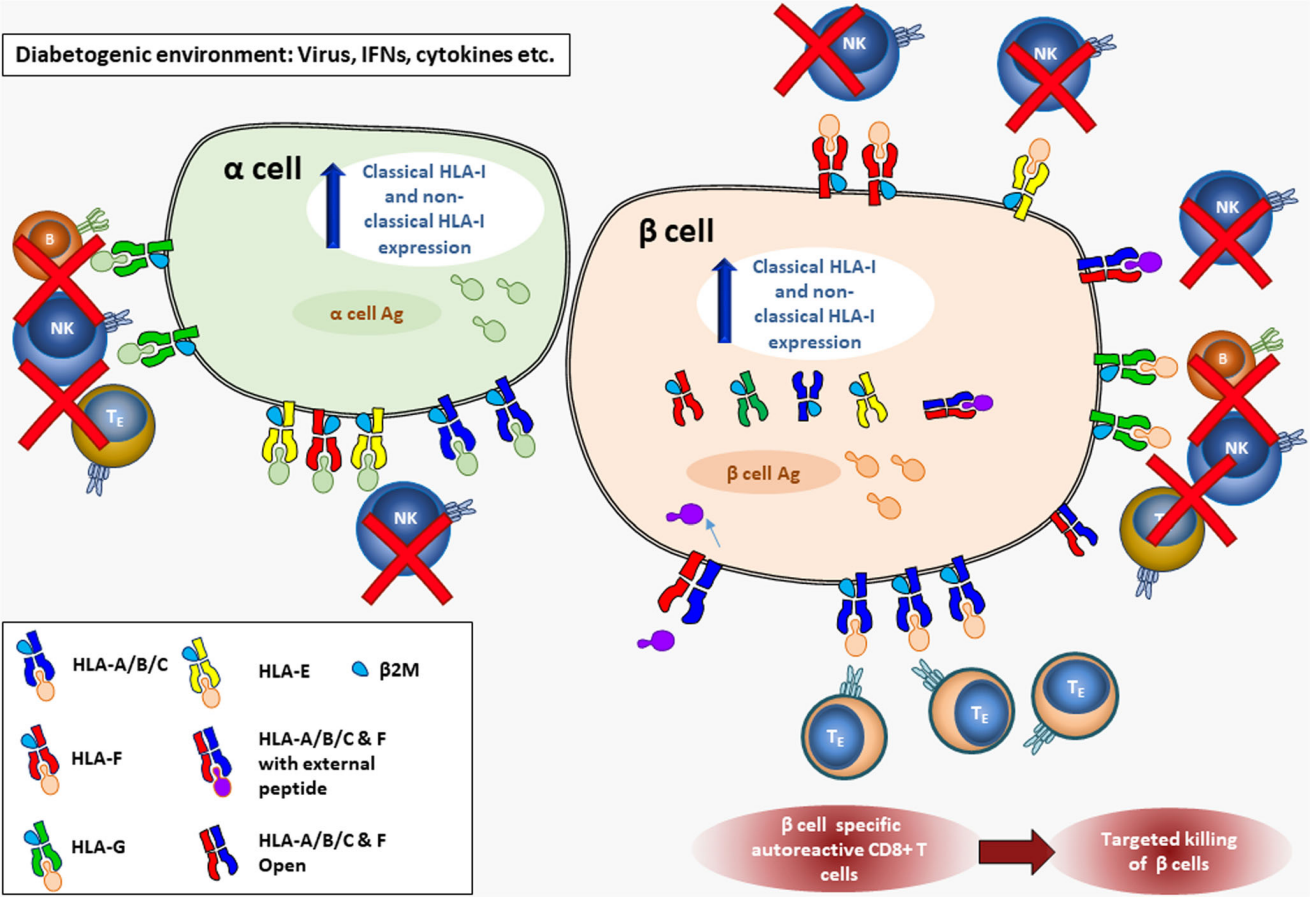
Model of the immunomodulatory impact of non-classical HLA class I in type 1 diabetes. Upregulation of classical and non-classical HLA-I expression in type 1 diabetes occurs on both β and α cells, which could impact on the activation and function of immune cells infiltrating into the islet as a result of an as yet unknown diabetogenic stimuli (e.g. viruses, interferons, cytokines). This likely also results in the presentation of α and β cell-derived peptides on their respective cells. Infiltrating islet-reactive CD8+ T cells will target only β cells presenting appropriate diabetes-associated peptides. The upregulation of non-classical HLA-I molecules will broadly have an inhibitory effect on NK cells through differential mechanisms. For example, HLA-E negatively regulates infiltrating NK cells through interactions with CD94/NK2 receptors, or HLA-F impacts NK cell activity via interaction with inhibitory receptors (such as ILT2, ILT4 and KIR). HLA-G can also negatively regulate NK, B and T cell function. The unique surface localisation of HLA-F in type 1 diabetes could reflect a yet unknown function, potentially facilitating the binding of exogenous peptides, which, once internalised, are presented via the classical HLA-I pathway resulting in the cross-presentation of peptides within β cells

In summary, the expression of HLA-E is induced by many of the same stimuli that regulate classical HLA-I, and the critical peptides presented by HLA-E are the leader peptides derived from these. As one of the key functions of HLA-E is to regulate NK cell activity, it is conceivable that the coordinated upregulation of HLA-E with classical HLA-I is designed to provide the target cell with protection from NK-mediated cytotoxicity, while still facilitating the ability of the CD8+ T cells to specifically kill any cells in which they recognise their target peptide bound to the classical HLA-I. In an inflammatory environment where many immune cells have been recruited, this mechanism would result in a shielding of bystander cells from immune attack, while still allowing the targeted destruction of select dysfunctional/virally infected cells (Fig. 1).

### HLA-F

HLA-F is highly conserved with low levels of polymorphism in humans: 38 alleles give rise to 6 different full-length protein molecules (according to the IMGT/HLA Database http://hla.alleles.org/ April 2019). Expression of HLA-F is tightly controlled and tissue-specific, with higher levels in lymphoid cells compared with non-lymphoid cells. Transcription can be upregulated by NFκB through EnhA, IFNγ via the ISRE and CIITA [14]. Although this molecule is known to associate with β_2_M and TAP, it is largely intracellular rather than expressed at the surface [27]. Upon immune cell activation, HLA-F can bind HLA-I heavy chains and facilitate their migration to the cell surface in an open-conformation (i.e. not bound to peptides) [28]. These complexes can act as ligands for inhibitory NK cell receptors to modulate the immune system [29]. HLA-F is also a genetic determinant of fecundability [30] and exerts an immunomodulatory function in maternal-foetal tolerance during pregnancy. Extravillous trophoblast cells that invade the maternal endometrial decidua express HLA-F, along with HLA-E and HLA-G, [31]. HLA-F is believed to interact with the inhibitory immunoglobulin-like transcript 2 (ILT2) and 4 (ILT4) receptors present on a variety of immune cells [32], as well as with KIR receptors present on NK cells [29] (Fig. 1). HLA-F also acts as a ligand for inhibitory KIR3DL2 to prevent astrocyte toxicity towards motor neurons in the development of amyotrophic lateral sclerosis [33].

Until recently, efforts to characterise HLA-F associated with β_2_M and sequence peptides have been largely unsuccessful [28, 29]. A more recent study, however, successfully engineered β_2_M to HLA-F as a single polypeptide in order to solve the first crystal structures of HLA-F and characterise peptide repertoires presented by the molecule. This showed that HLA-F can exist as an open conformer but can also bind a diverse number of peptides (> 2000) between 7 and 30 residues long and be recognised by leukocyte immunoglobulin-like receptor 1 (LIR1). This raises the possibility that HLA-F could be recognised by other immune receptors and elicit different responses depending on their conformation [34••, 35]. Although most immunomodulatory actions of HLA-F are inhibitory, under some circumstances HLA-F open conformers activate primary human NK cells. For example, HLA-F binding to the KIR3DS1 receptor can activate NK cells and elicit an antiviral response to inhibit HIV-1 replication [36].

### HLA-G

HLA-G is the most polymorphic of the non-classical isoforms, but its grade of polymorphism is still low (61 alleles encode 19 proteins according to the IMGT/HLA Database http://hla.alleles.org/ April 2019) compared to classical HLA-I, for which > 4000 alleles encode thousands of proteins. Alternative splicing of the primary mRNA leads to 7 isoforms of HLA-G. G1–G4 isoforms have transmembrane and cytoplasmic domains, meaning they are membranous, whereas G5–G7 isoforms are soluble. G1 and G5 are also able to bind non-covalently to β_2_M [11]. Unlike HLA-E and the classical HLA-I molecules, HLA-G is not constitutively expressed in tissues, but it is found in a limited number of cell types, such as cytotrophoblast cells at the maternal-foetal interface of the placenta, corneal, nail matrix, embryonic mesenchymal stem cells and pancreatic islet β cells [37, 38]. HLA-G can bind a repertoire of self-peptides. Regulation of HLA-G transcription is peculiar, as this gene is unresponsive to NFκB, IRF1 and CIITA mediated pathways, although it may be responsive to NLRC5 [12, 14]. Alternative regulatory elements have been described, some examples of which include a heat shock element (HSE), which would respond to stress-induced heat shock proteins (HSP), long interspersed elements (LINEs) and the hypoxia-inducible factor (HIF), involved in cellular responses to oxygen depletion [39]. HLA-G mediates immune responses of NK, B and T cells through interactions with inhibitory receptors including ILT-2, ILT-4, KIR2DL4 and CD160 (Fig. 1). It can inhibit a wide range of immune functions, including the antigen-specific cytolytic function of cytotoxic T cells, alloproliferative response of CD4+ T cells, ongoing proliferation of NK and T cells and maturation of dendritic cells [11, 40]. Hence, HLA-G upregulation in tumours and in the placenta during pregnancy is advantageous for concealment from immune surveillance and to impart tolerance. Interestingly, a recent study showed that HLA-E complexed with an HLA-G leader peptide enrich a population of adaptive NK cells [41]. These cells, characterised by a reduced FεCRγ expression, have an upregulated CD25 expression, increased proliferation and increased antibody-dependent cell-mediated cytotoxicity and prompt a heightened IFNγ response. NK cells exposed to HLA-E molecules presenting HLA-C leader peptides, in contrast, did not show the same effects [41].

## What Is Known About Non-classical HLA Class I in Type 1 Diabetes?

### HLA-E

**Fig. 2:**
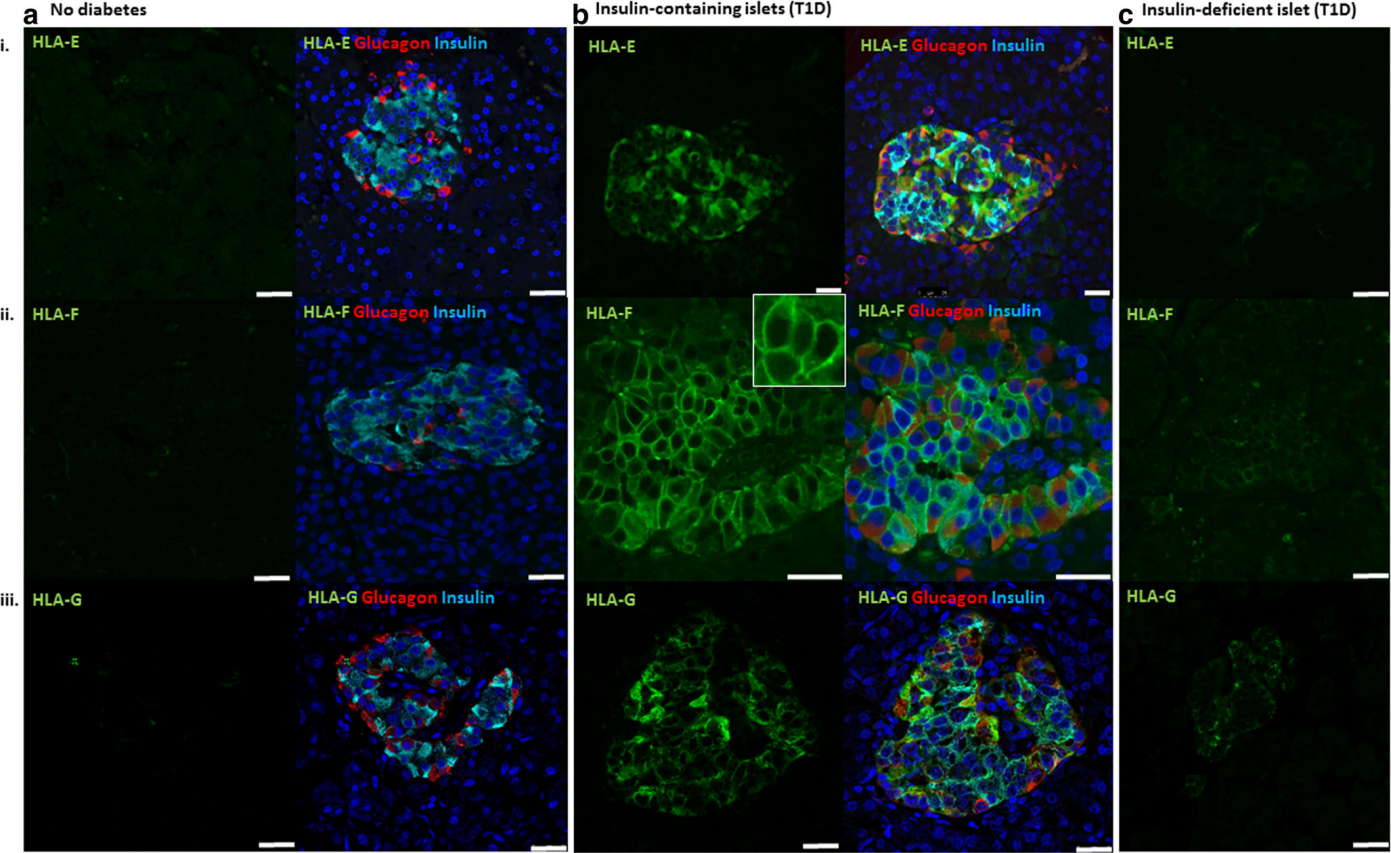
Non-classical HLA class I molecules are elevated in the islets of patients with type 1 diabetes. Representative immunofluorescence micrographs depicting the expression of (i) HLA-E, (ii) HLA-F and (iii) HLA-G in pancreatic islets. Samples from control individuals (**a**) and from patients with type 1 diabetes with **b** insulin-containing islets and **c** insulin-deficient islets were immunostained for non-classical HLA-I (green), glucagon (red), insulin (light blue) and DAPI (dark blue). The surface expression of HLA-F is demonstrated in the magnified inset (white box). Scale bar 25 μm

Very few studies have directly examined the expression of HLA-E in the pancreas. However, studies utilising the unique type 1 diabetes pancreas resection material from recently diagnosed patients enrolled in the Norwegian DiViD study [42] demonstrated that HLA-E RNA expression was elevated in inflamed islets when compared with islets from control pancreata. HLA-E expression was particularly high in the non-infiltrated islet core of the type 1 diabetes donors when compared to the peri-islet area that contained infiltrating immune cells, which suggests that the islet cells rather than the immune cells have the highest expression [43••]. Elevated HLA-E RNA expression was also observed in 4 further recent-onset T1D organ donors, and elevated protein was confirmed in select islets of one of these donors. Hyperexpression was also observed in exocrine tissue of the same T1D donor [44]. More recent studies of recent-onset T1D donors from the Exeter Archival Diabetes Biobank (EADB) and DiViD have revealed that HLA-E is specifically upregulated in the ICIs. This was confirmed at the RNA and protein level. Intriguingly, HLA-E expression appears higher in α cells but was also observed in β cells. HLA-E levels in insulin-deficient islets are comparable with those of islets in non-diabetic controls (Fig. 2; Richardson, unpublished data). There is limited evidence to suggest that certain HLA-E genotypes (HLA-E107R/G) are associated with age at onset of type 1 diabetes [45].

### HLA-F

Upregulation of HLA-F (at both the RNA and protein level) has been observed in the insulin-containing islets of patients with recent-onset type 1 diabetes when compared with islets from non-diabetic controls [16••]. This upregulation is lost when islets are devoid of insulin, suggesting that like the classical HLA-I, the stimulus regulating its expression is derived either from the β cells themselves, or requires the presence of β cells to elicit the release of this factor from other cell types, e.g. infiltrating immune cells. Similar observations were made in donor samples from the nPOD, DiViD and EADB cohorts. These findings were supported further by RNA expression data, which showed that HLA-F was upregulated by 1.71 ± 0.04-fold in islets from patients of the DiViD cohort [16••]. Furthermore, bulk sorted β cells from T1D donors showed increased expression of HLA-F compared to non-diabetic donors [15].

Similar to classical HLA-I in T1D, HLA-F was expressed in both α and β cells. Uniquely, in contrast with conventional HLA-F expression, this molecule was primarily localised to the surface of the cell (Fig. 2; inset), co-localising with classical HLA-I [16••]. The ability of HLA-F to aid the translocation of free HLA-I to the cell surface, where it may bind to exogenous peptides, raises the possibility that cross-presentation of peptides from within the extracellular space could be occurring in α and β cells in T1D. Proteins released from neighbouring β cells may be bound, endocytosed and processed within a cell, and subsequently presented in the context of normal HLA-I, which could have important implications for immune responses at the islet site [16••] (Fig. 1).

### HLA-G

Associations between an HLA-G polymorphism and the age of onset of type 1 diabetes have been found [46]. Homozygosity for the deletion of 14 bp of the 3′ untranslated region of HLA-G was associated with an earlier age of type 1 diabetes onset, whereas heterozygotes (carrying only one deletion) had a later age of onset of type 1 diabetes [46]. HLA-G is expressed by pancreatic islet and duct cells, and it is upregulated in response to pro-inflammatory cytokines [37]. In pancreatic endocrine cells, HLA-G is constitutively expressed at low levels as a β_2_M-free heavy chain protein and remains mainly intracellular. Interestingly, HLA-G appears to be associated with a subset of insulin-containing granules and can be exported to the β cell surface, not only through the constitutive secretory pathway, but also through the regulated pathway by which insulin is secreted [37]. Numerous autoantigens in islet immunity are components of secretory granules [47–49]; therefore, insulin exocytosis sites may be the sites where the immunogenic ligands become exposed. As the activation of autoreactive T cells depends upon surface density of antigen/MHC complexes, this may lead to the activation of low-affinity cytotoxic T cells. Consequently, the presence of HLA-G at such granule exocytosis sites could represent a prevention mechanism for aberrant immune activations.

In pancreas tissue from patients with recent-onset type 1 diabetes, upregulation of HLA-G at the protein level can be seen in the islets, in both α and β cells. Expression is predominantly cytoplasmic, but HLA-G is also observed at the cell surface (Fig. 2; Wyatt, unpublished data).

## Virus Manipulation of Non-classical HLA-I

A viral etiology for the development of type 1 diabetes has long been hypothesised and has been a topic of great controversy [50–57, 58••]. At least 10 viruses have been reported to be associated with the development of type 1 diabetes-like syndromes in animals [59]. Some of them, such as encephalomyocarditis (EMC) virus in mice, are β-cell-tropic: these viruses can determine either acute widespread destruction of β cells, in the presence of high viral titer, or initial infection of β cells followed by recruitment of immune cells, islet inflammation and β cell loss [59]. Other viruses, such as Kilham rat virus, lead to preferential activation of effector T cells, thus facilitate autoimmune responses and depletion of β cells [59]. While epidemiologic evidence exists in humans, suggesting a role of viral pathogens in type 1 diabetes development and progression, there has been debate in the field, as no definitive causative agent has been identified. This is connected to the fact that the detection of viruses in patients has been more than sporadic, but not truly conclusive. Nevertheless, there is evidence for the role of viruses (particularly enteroviruses) in type 1 diabetes and other auto-immunities [42, 52, 56, 60–63].

Viruses have evolved a range of mechanisms to escape host immune recognition and innate or adaptive immune responses, some of which are based on the hijacking of non-classical HLA-I functions. Viruses such as Epstein-Barr virus (EBV), cytomegalovirus (CMV), parvovirus-B19 (Parvo B-19), herpes simplex virus type 1 (HSV-1) and RABV26 modulate HLA-E and HLA-G, facilitating the immune escape of infected cells [54, 64–72]. HSV-1, a neuronotropic virus with potential for acute infection and neuron latency, and rabies virus (RABV), a neuronotropic virus triggering acute neuron infection, both upregulate the neuronal expression of several HLA-G isoforms [73]. Herpes B virus, a simian virus, stimulates an upregulation of HLA-G and HLA-E, which probably facilitates immune escape of the infected cells [74]. Hepatitis C virus (HCV) and Japanese encephalitis virus (JEV) can increase expression of HLA-F [75, 76].

Maternal infections during pregnancy are associated with a doubling of the risk of type 1 diabetes in the offspring [63]. Haplotypes and polymorphisms, such as the 14-bp deletion/insertion polymorphism in the 3′ untranslated region of HLA-G, could be involved in the mother-to-child vertical transmission of viruses, such as in the case of HIV [77–79].

The upregulation in the expression levels of HLA-E, HLA-F and HLA-G in insulin-containing islets of patients with type 1 diabetes could conceivably be linked to a viral infection where the virus is attempting to shield itself from the immune system.

## Conclusions and Future Perspectives

This review highlights emerging evidence of the involvement of non-classical HLA-I molecules in type 1 diabetes. Expression of HLA-E, HLA-F and HLA-G are all elevated in the insulin-containing islets of patients with type 1 diabetes and while expression was not restricted to β cells, the presence of β cells was a requirement for the elevated expression. In a pattern that mirrors that of classical HLA-I, non-classical HLA-I hyperexpression is lost in islets that are devoid of β cells (Fig. 2). Upregulation of these molecules may represent a protective response to inflammatory mediators, such as interferons, present in the microenvironment of islets with residual β cells. As non-classical HLA class I molecules most frequently impart inhibitory signals to immune cells, particularly to NK cells, their expression by islet cells could be a defense mechanism from immune cells recruited to the islet during the inflammatory process (Fig. 1). Several small studies have identified polymorphism of HLA-E and HLA-G alleles that are associated with younger age of type 1 diabetes onset. As such, the ability of an individual to activate these inhibitory pathways could determine how rapidly β cells are destroyed during an autoimmune attack. Alternatively, this observed upregulation may be connected to viral infections and viral mechanisms of immune escape. Further investigations to delineate the causes and implications of non-classical HLA-I hyperexpression in type 1 diabetes pancreatic islets are, therefore, warranted.
